# Air Pollution and Its Adverse Effects on the Central Nervous System

**DOI:** 10.7759/cureus.38927

**Published:** 2023-05-12

**Authors:** Ameerah Ruzeeq Alhussaini, Meaad Refaay Aljabri, Zeyad T Al-Harbi, Gadah Abdulrahman Almohammadi, Talal M Al-Harbi, Shahid Bashir

**Affiliations:** 1 College of Medicine, Taibah University, Madinah, SAU; 2 College of Medicine, Imam Abdulrahman Bin Faisal University, Dammam, SAU; 3 Neurology, King Fahad Specialist Hospital, Dammam, SAU; 4 Neuroscience, Neuroscience Center, King Fahad Specialist Hospital, Dammam, SAU

**Keywords:** stroke, neuropathology, neuroinflammation, central nervous system disorder, air pollution

## Abstract

Air pollution is recognized as a significant public health problem and is associated with illnesses of the central nervous system (CNS) as well as neuroinflammation and neuropathology. Air pollution may cause chronic brain inflammation, white matter abnormalities, and microglia activation, which increases the risk of autism spectrum disorders, neurodegenerative disorders, stroke, and multiple sclerosis (MS).

Methods: A literature review was done on “PubMed, EMBASE and Web of Science” on the relationship of air pollution with MS and stroke, using the keywords “air pollution” OR “pollution”; “ambient air pollution,” “particulate matter, ozone, black carbon” AND “stroke” OR “cerebrovascular diseases,” “multiple sclerosis,” “neuroinflammation,” or “neurodegeneration.”

Results: We first identified 128 articles and their related websites, of which 44 articles were further selected for analysis mainly based on study relevance, study quality and reliability, and date of publication.

Further studies on air pollution and its adverse effects on the CNS are needed. The findings of such studies will support the development of appropriate preventive measures in the future.

## Introduction and background

The World Health Organization (WHO) estimates that ambient and indoor air pollution cause over 4.2 and 3.8 million deaths each year, respectively [1]. It is well documented that air pollution increases adverse health effects related to cardiovascular and respiratory diseases [2,3]. Recently, growing evidence has linked air pollution to illnesses of the central nervous system (CNS) as well as to neuroinflammation and neuropathology [2-4].

Air pollution can be defined as any process that introduces particles into the atmosphere that can cause harm to living organisms and the environment [5]. Common forms of air pollution consist of components derived from various natural and anthropogenic sources, including carbon monoxide (CO); sulfur oxides (SOx); particulate matter (PM); nitrogen oxides (NOx); ozone (O3); methane; and other gases, metals, and volatile organic compounds [3].

PM is the most widely distributed type of air pollution and has been linked to a variety of diseases [2]. The Environmental Protection Agency (EPA) classifies these particles according to their expected lung penetration capacity. There are two categories: (i) coarse particulate matter (PM10) has an aerodynamic diameter of 10 μm, and (ii) fine part particulate matter (PM2.5) has an aerodynamic diameter of 2.5 μm. There are many sources for both types of PM, including road dust, agricultural dust, riverbeds, construction sites, and mining operations [5].

A number of recent studies on human epidemiology and animal toxicology have raised concerns about the potential negative impact of air pollution on the CNS. Air pollution may cause chronic brain inflammation, white matter abnormalities, and microglia activation, which increases the risk of autism spectrum disorders, neurodegenerative disorders (e.g., Alzheimer’s disease (AD) and Parkinson’s disease (PD)), stroke, and multiple sclerosis (MS) [3].

The aim of this literature review is to summarize the findings of previous studies on air pollution and its association with CNS diseases, focusing on MS and stroke.

## Review

Method

A literature review was conducted using PubMed, EMBASE, and Web of Science. A search was conducted for articles published on the relationship of air pollution with MS and stroke, using the keywords “air pollution” OR “pollution”; “ambient air pollution,” “particulate matter, ozone, black carbon” AND “stroke” OR “cerebrovascular diseases,” “multiple sclerosis,” “neuroinflammation,” or “neurodegeneration.” A total of 44 articles [2-53] were collected for the analysis based on study type, abstract, quality, reliability, and publication date (Table 1).

**Table 1:** The impact of air pollution on the multiple sclerosis and stroke.

Author	Participants	Main result		Study
Costa et al., 2014 [2]	-	Exposure to air pollution has been associated with increased expression of neurodegenerative disease pathologies markers.	Multiple Sclerosis	Neurotoxicants Are in the Air: Convergence of Human, Animal, and In Vitro Studies on the Effects of Air Pollution on the Brain
Heydarpour et al., 2014 [8]	N=2188	The exposure to NO_2_, NOx, PM_10_, and SO_2_ for long-term is risk factor for MS.		Potential Impact of Air Pollution on Multiple Sclerosis in Tehran, Iran
Lavery et al., 2018 [9]	MS cases (N=290) and healthy controls (N=442)	Significant association between air pollutants (CO, SO2 and lead) and higher odds for MS in pediatric.		Urban air quality and associations with pediatric multiple sclerosis
Gregory II et al., 2008 [10]	N=9,072,576	A potential role of PM10 in MS etiology of MS in females.		Multiple Sclerosis disease distribution and potential impact of environmental air pollutants in Georgia
Angelici et al., 2016 [11]	N=8287	Exposure to PM10 have a role in determining MS occurrence and relapses.		Effects of particulate matter exposure on multiple sclerosis hospital admission in Lombardy region, Italy
Bergamaschi et al., 2018 [12]	N=52	Air pollution causes inflammatory exacerbations which may make it a risk factor for MS.		Air pollution is associated to the multiple sclerosis inflammatory activity as measured by brain MRI
Jeanjean et al., 2018 [13]	N=424	In the single-pollution model, significant associations between the exposures to air pollutants (PM10, NO2, and O3) and the relapses in MS. In the multi-pollutant model, there is a significant association between O3 and the relapses in MS.		Ozone, NO2 and PM10 are associated with the occurrence of multiple sclerosis relapses. Evidence from seasonal multi-pollutant analyses
Mehrpour et al., 2013 [14]	N=174	Air pollutants are potential risk factors for relapse in MS.		Effect of Air Pollutant Markers on Multiple Sclerosis Relapses
Oikonen et al., 2003 [15]	N=1,205	A high level of ambient air PM10 may enhance the occurrence of the seasonal changes in MS relapse.		Ambient air quality and occurrence of multiple sclerosis relapse
Roux et al., 2017 [16]	N=536	A positive association between the exposure to PM10 and the risk of MS relapse in cold season.		Air pollution by particulate matter PM 10 may trigger multiple sclerosis relapses
Vojinović et al., 2015 [17]	N=101	The decrease in the numbers of days with low air pollution during the time of low vitamin D specifically while increasing the cloudiness, increase the risk of relapses in MS in southern continental parts of Europe.		Disease relapses in multiple sclerosis can be influenced by air pollution and climate seasonal conditions
Ashtari et al., 2018 [18]	N=1170	Air pollution was related to the MS expanded disability status scale (EDSS), severity, and remission of MS disease.		An 8-year study of people with multiple sclerosis in Isfahan, Iran: Association between environmental air pollutants and severity of disease
Tateo et al., 2018 [19]	N=1435	A strong association between the exposure to PM2.5 and the prevalence of MS.		PM2.5 levels strongly associate with multiple sclerosis prevalence in the Province of Padua, Veneto Region, North-East Italy
Bergamaschi et al., 2021 [20]	N=927	The risk of MS is low in individuals living in rural areas where the level of PM_2.5_ is low.		PM2.5 exposure as a risk factor for multiple sclerosis. An ecological study with a Bayesian mapping approach
Türk Börü et al., 2020 [21]	From Eregli: 32261 From Devrek: 21963	As compared to the rural city, the MS prevalence rate is more than double in the area home to an iron and steel factory which suggest that air pollution is a potential MS etiological factor.		Air pollution, a possible risk factor for multiple sclerosis
Yuchi et al., 2020 [22]	N=678,000	No association between air pollutants and the incidence of multiple sclerosis.		Road proximity, air pollution, noise, green space and neurologic disease incidence: a population-based cohort study
Bai et al., 2018 [23]	N=2,824,478	No association between long-term exposures to PM_2.5_, O_3_, and NO_2_, and MS incidence in adults.		Long-term exposure to air pollution and the incidence of multiple sclerosis: A population-based cohort study
Palacios et al., 2017 [24]	Study1: 121,700 Study2: 116,671	No association between the exposure to PM air pollution and the risk of MS.		Exposure to particulate matter air pollution and risk of multiple sclerosis in two large cohorts of US nurses
Chen et al., 2017 [25]	N=9247	No association between the MS incidence and living near to heavy traffic.		Living near major roads and the incidence of dementia, Parkinson's disease, and multiple sclerosis: a population-based cohort study
Cortese et al., 2020 [27]	N=57 MS patients and 19 healthy controls	In MS patients the exposure to PM10 induce the autoreactive Th17 lymphocytes production in the lung and enhance their migration through the blood-brain barrier.		Air pollution as a contributor to the inflammatory activity of multiple sclerosis
Niu et al., 2021 [29]	23 million participants (conducted from 68 studies)	There is a positive association between air pollution exposure and the increase in the risk of stroke incidence (SO2, PM2.5, and NO2), hospital admission (PM10, PM2.5, NO2, SO2, O3, and CO), and mortality (PM10, PM2.5, NO2, and SO2).	Stroke	Association between exposure to ambient air pollution and hospital admission, incidence, and mortality of stroke: an updated systematic review and meta- analysis of more than 23 million participants
Guo et al., 2017 [30]	N=95562	A borderline significant association was observed between the exposure to NO2 modeled as an averaged lag effect and the risk of ischemic stroke.		Ambient Air Pollution and Risk for Ischemic Stroke: A Short-Term Exposure Assessment in South China
Korek et al., 2015 [31]	N=20.070 (868 stroke cases)	NO_x _and PM_10 _from local traffic are associated with the incidence of stroke in a region with comparatively low air pollutant levels.		Traffic-related air pollution exposure and incidence of stroke in four cohorts from Stockholm
Lisabeth et al., 2018 [32]	N=3508 (ischemic stroke= 2350, TIA=1158).	Borderline significant association between PM_2.5 _and O_3 _exposure and ischemic stroke/TIA.		Ambient Air Pollution and Risk for Ischemic Stroke and Transient Ischemic Attack.
Zhang et al., 2021 [33]	N=109,975	Elevated levels of PM10, PM2.5, NO3, CO, and SO2 positively associated with the increase in TIA hospital admissions.		Association between short-term exposure to ambient air pollution and hospital admissions for transient ischemic attacks in Beijing, China
Shin et al., 2019 [34]	N=5071956	PM_2.5_, NO_2_, O_3 _and O_X _consistently associated with higher incidence for stroke hospitalization. With the exception of NO_2 _all other pollutants were associated with ischemic stroke more than hemorrhagic stroke.		Ambient Air Pollution and the Risk of Atrial Fibrillation and Stroke: A Population Based Cohort Study.
Amini et al., 2020 [35]	N=23,423	PM_2.5_ was significantly associated with overall stroke, stronger with ischemic stroke than hemorrhagic stroke.		Long-term exposure to air pollution and stroke incidence: A Danish Nurse cohort study
Huang et al. 2019 [37]	N=117575	Every 10 µg/m^3^ increase in PM_2.5 _level was associated with increased risk of stroke incidence, increased ischemic stroke incidence and increased in hemorrhagic stroke incidence.		Long term exposure to ambient fine particulate matter and incidence of stroke: prospective cohort study from the China-PAR project
Wellenius et al., 2005 [38]	N=174817	PM_10_ was positively associated with stroke admission on the same day, ischemic stroke rather than hemorrhagic stroke.		Air Pollution and Hospital Admissions for Ischemic and Hemorrhagic Stroke Among Medicare Beneficiaries
Zhang et al., 2018 [39]	N=48,122	The increase in ambient PM_2.5 _by 10 μg/m^3^ was associated with the increase of ischemic and hemorrhagic stroke mortality.		Acute Effects of Particulate Air Pollution on Ischemic Stroke and Hemorrhagic Stroke Mortality
Wellenius et al., 2012 [40]	N=1705	PM_2.5_ exposure increased the risk of ischemic stroke and the greatest risk was within 12-14 hours of exposure.		Ambient Air Pollution and the Risk of Acute Ischemic Stroke
Qiu et al., 2017 [41]	N=61447	PM_2.5_ had a statistically significant association with ischemic stroke and unspecified stroke and no association with hemorrhagic stroke.		Fine particulate matter exposure and incidence of stroke A cohort study in Hong Kong.
Lin et al., 2016 [42]	N=5.5 million	PM_10_, PM_2.5_, and PM_1_ were significantly associated with stroke mortality and related to hemorrhagic stroke rather than ischemic stroke.		Differentiating the effects of characteristics of PM pollution on mortality from ischemic and hemorrhagic strokes.
Ljungman et al., 2019 [43]	N=114,758	BC exposure from traffic source is significantly associated with stroke incidence. PM_10_ and PM_2.5 _were not associated with stroke incidence.		Long-Term Exposure to Particulate Air Pollution, Black Carbon, and Their Source Components in Relation to Ischemic Heart Disease and Stroke.
Sun et al., 2019 [44]	N=5417 (ischemic=4300, hemorrhagic=924, undetermined type=193)	NO_2_ and NO_X_ were associated with higher relative risk of hemorrhagic stroke. The association was mor pronounced among non-obese participants. PM_2.5 _and PM_10 _were not associated with stroke.		Short-term Exposure to Air Pollution and Incidence of Stroke in the Women’s Health Initiative
Vivanco-Hidalgo et al., 2019 [45]	N=2761	No association between PM2.5 and initial stroke severity. Residential with higher greenspace surrounding was associated with less severe acute ischemic stroke.		Association of residential air pollution, noise, and greenspace with initial ischemic stroke severity.
Vivanco-Hidalgo et al., 2018 [46]	N=3311	PM_2.5 _and BC were not associated with acute ischemic stroke, but in subtype analysis BC was associated with higher risk of ischemic stroke symptoms onset due to large artery atherosclerosis.		Short-term exposure to traffic-related air pollution and ischemic stroke onset in Barcelona, Spain
Han et al., 2015 [47]	N=3001 (2202 were IS and 799 were ICH)	There is a strong positive correlation between NO_2_ and the incidence of intracerebral hemorrhage among the older age group.		Effect of Seasonal and Monthly Variation in Weather and Air Pollution Factors on Stroke Incidence in Seoul, Korea
Tang et al., 2019 [48]	N=1646	One-pollutant model: every 10 µg/m^3^ increase in O_3_ had significant impact on daily emergency outpatient visits for acute stroke, greater change in males, increased risk for those who aged > 60 years and in the group with pre-existing HTN. Two-pollutant model: the combination between O_3_ and 10 µg/m^3^ increase in NO_2_ increased the risk of emergency stroke.		Short‑term exposure to air pollution and occurrence of emergency stroke in Chongqing, China
Henrotin et al., 2007 [49]	N=2078	Single pollutant model: O_3_ levels is associated with ischemic stroke in men over 40 years old, no significant association with hemorrhagic stroke. Two-pollutant model: significant O_3_ effect with other pollutant particularly PM_10_.		Short-term effects of ozone air pollution on ischemic stroke occurrence: a case-crossover analysis from a 10-year population-based study in Dijon, France.
Suissa et al., 2013 [50]	N=1729	An increase in O_3_ level by 10 µg/m^3 ^is associated with increased risk of recurrent stroke and large artery stroke.		Ozone air pollution and ischemic stroke occurrence: a case-crossover study in Nice, France.
Chen et al., 2020 [51]	N=276,736	Compared with stroke without hypertension, greater risk of hospitalization for stroke with hypertension caused by SO2 and NO2, with lower risk observed due to O3. For stroke patients with coronary atherosclerosis, O3 and SO2 appeared to be protective.		Effect of air pollution on hospitalization for acute exacerbation of chronic obstructive pulmonary disease, stroke, and myocardial infarction
Andersen et al., 2014 [52]	N=52215	Borderline significant association between NO_2_ and (stroke incidence, and stroke hospitalization). The association was for the ischemic and unspecified stroke, no association with hemorrhagic stroke.		Stroke and Long-Term Exposure to Outdoor Air Pollution from Nitrogen Dioxide A Cohort Study.
Huang et al., 2017 [53]	N=147624	O_3_ and stroke admission were positively associated during warm season and negatively associated during cold season. Both SO_2_ and NO_2 _were positively associated with stroke admission especially in those aged < 65 years and during warm season.		Gaseous Air Pollution and the Risk for Stroke Admissions: A Case-Crossover Study in Beijing, China

Results and discussion

Multiple Sclerosis

MS is a chronic inflammatory autoimmune neurological disorder that impacts the CNS [6]. In recent decades, the prevalence of MS has increased; today, around 2.2 million individuals suffer from MS worldwide [7]. Air pollution has been implicated as a chronic environmental cause of neuroinflammation, reactive oxygen species (ROS), and neuropathology, all of which can contribute to CNS disorders [2,4]. Older and very young individuals seem to be particularly susceptible to neurotoxicity induced by air pollution. Exposure during the prenatal or postnatal period may contribute to behavioral abnormalities and developmental disabilities, while older people may develop neurodegenerative diseases due to exposure to air pollution [4]. Heydarpour et al. suggest that long-term exposure to air pollutants may be a risk factor for MS; this conclusion is due to an observed significant difference between the exposure of MS patients and controls to NO2, NOx, PM10, and SO2 [8]. A case-control study of the association between air pollutants and pediatric MS found that higher exposure to air pollutants (CO, SO2, and lead) was significantly associated with a higher risk of MS in pediatric patients [9].

Several studies of adult MS have found that an increase in PM10 exposure is associated with an increased risk of MS in adults [8,10,11], active MS inflammatory lesions detected by magnetic resonance imaging (MRI), and MS relapse [11-16]. A strong association has been observed between active MS inflammatory lesions (shown in an MRI) and a higher concentration of PM10; for every increase of PM10 by an increment of 30 µg/m^3^, the risk of an inflammatory lesion increases by 86% [12].

A deeper investigation of various air pollutants revealed that the risk of MS relapse increased following exposure to NO2 and PM10 during cold seasons and that exposure to O3 increases the risk of relapse during hot seasons [17]. Vojinović et al. found a significant negative correlation between seasonal vitamin D levels (which are higher from July to October in the northern hemisphere and MS relapse). The findings of this five-year observational study confirmed the influence of seasonal conditions and air pollution on MS relapse risk [17,18]. Air pollution has also been found to correlate with poorer scores on the MS expanded disability status scale (EDSS), lower rates of MS remission, higher MS severity, and poorer recovery from the first MS event [18].

An Italian study in the northeastern province of Padua (Veneto region) found a higher prevalence of MS in urban areas, which have higher PM2.5 levels than rural areas [19]. Similarly, Bergamaschi et al. suggest that the risk of developing MS is reduced as air pollution reduces [20]. A recent study in Turkey found that MS is more than twice as prevalent in regions near steel and iron factories than in rural areas [21]. These findings support the hypothesis that air pollution may play a role in the etiology of MS.

However, the results of several other studies are inconsistent with this hypothesis. A recent study in Canada found no association between air pollutants and MS incidence [22]. Other studies have found no link between MS risk and PM2.5 [9,22,23], PM10 [9,24], NO2, or O3 [23]. Chen et al. also found no association between living close to heavy traffic and MS incidence [25].

Several theories have been proposed regarding the impact of air pollutants on the CNS and MS risk. First, respiratory exposure to air pollutants may trigger oxidative stress and increase the permeability of the epithelial wall, resulting in the release of pro-inflammatory cytokines and provoking an immune response by activating the aggressive auto-reactive T cells and enhancing their migration to the CNS through the blood-brain barrier (BBB) [12,21,26,27]. PM10, in particular, may play a pro-inflammatory role by upregulating the expression of C-C chemokine receptors 6 (CCR6) on circulating cluster of differentiation four (CD4+) T cells and provoking the production of T helper 17 cells (Th17) polarizing cytokines in cells that regulate innate immunity [27]. A second theory proposes that inhaled particles may be translocated to the CNS through the olfactory system [26]. A third explanation is that exposure to air pollutants, along with lifestyle changes, may interrupt the normal balance between self-tolerance (the unresponsiveness state of the immune system to self-antigens [28]) and immunity [18]. Other probable mechanisms include insufficient vitamin D, which may be an indirect effect of exposure to air pollution [12,18]. MS risk could also be related to genetics, particularly epigenetic modifications, and, more specifically, DNA methylation alterations [26].

Stroke

A stroke is an acute disturbance of cerebral circulation caused by arterial stenosis, blockage, or rupture in patients with cerebrovascular disease; it may occur due to a variety of inducing factors [29]. It is the second leading cause of death globally and a significant cause of hospitalization, long-term disability, and high medical costs [30]. The global burden of stroke is enormous and growing, especially in developing countries [30].

Particulate Matter ​​​​​and Stroke

Several studies have found an association between PM and stroke. Long-term exposure to PM10 from local traffic was associated with the incidence of stroke in a region with comparatively low air pollution levels [31]. Another study found a borderline significant association between same-day and previous-day exposure to PM2.5 exposure and the risk of stroke or transient ischemic attack (TIA) [32]. Elevated PM10 and PM2.5 levels have also been positively associated with an increase in hospital admissions due to TIA [33]. Another study found that PM2.5 was associated with a higher incidence of hospitalization due to stroke; this association was stronger for ischemic than hemorrhagic stroke. Moreover, individuals with lower incomes had a higher risk of stroke after exposure to PM2.5 [34]. Yet another study showed a positive association between long-term PM2.5 exposure and stroke incidence, particularly ischemic stroke [35].

In one study, every 10μg/m^3^ increase in PM2.5 was associated with an increase of 0.69% in stroke morbidity. This changes to an increase of 0.80% in the risk of stroke morbidity for females and to an increase of 0.78% in the risk of stroke morbidity for individuals over 65 years old. No association was between stroke and PM10 or PMc [36]. Another study found that each 10 μg/m^3^ increase in PM2.5 was associated with a 20% increase in the incidence of ischemic stroke and a 12% increase in the incidence of hemorrhagic stroke. Ischemic stroke is more common in elderly people and people with normal weight [37]. One study also found a significant association between PM10 exposure and ischemic but not hemorrhagic stroke [38]. Similarly, another study showed that a 10 μg/m^3^ increase in PM2.5 was positively associated with an increase of 0.23% in mortality due to ischemic stroke and an increase of 0.37% in mortality due to hemorrhagic stroke. The same study found that exposure to PM10 increased the risk of mortality due to ischemic stroke by 0.16% but did not impact the risk of mortality due to hemorrhagic stroke [39].

Another study found that PM2.5 exposure increased the risk of stroke onset, particularly 12 to 14 hours after exposure. In that study, patients experienced ischemic stroke due to large artery atherosclerosis or small vessel occlusion rather than cardioembolism [40]. In another study, long-term exposure to PM2.5 was also associated with a higher incidence of ischemic and unspecified stroke; this association was strongest in older people, those with lower levels of education, and men who smoked. The association with hemorrhagic stroke was less clear [41]. In contrast, another study found that PM10, PM2.5, and PM1 were associated with hemorrhagic stroke but not ischemic stroke. That study also found an association between stroke mortality and secondary aerosol components of PM2.5, including sulfate, nitrate, and ammonium [42].

However, other studies have found no association between PM and stroke. For example, two studies found no association between stroke and PM2.5 and PM10 [43,44]. Another study found no association between initial stroke severity and long-term residential PM2.5 exposure [45]. Yet another found no evidence of an association between short-term PM2.5 exposure and the onset of stroke symptoms, including ischemic stroke, in the 72 hours following exposure [46]. Interestingly, another study showed that PM10 was linked to a lower incidence of ischemic stroke and a higher incidence of intracerebral hemorrhage; this risk increased for every 10 mg/m^3^ increase in PM10. However, after controlling for other variables, these relationships were found to be insignificant [47].

Ozone and Stroke

O3 is a strong oxidant that can affect the oxidant-antioxidant balance in the body, thereby contributing to ischemic cerebrovascular diseases [48]. In one study, a 10 μg/m^3^ increase in O3 was associated with emergency outpatient stroke in Chongqing City. This association was particularly strong for males, for whom the risk increased by 0.77% more than it did for females; the risk for individuals over 60 years old increased by 1.14%, and the risk for individuals with pre-existing hypertension increased by 0.26% [48].

Another study showed a significant association between O3 levels and ischemic stroke in men over 40 years old; this association was weaker in women [49]. A 10 µg/m^3^ increase in O3 was associated with an approximately 12% increase in the risk of stroke subtypes for recurrent stroke and with an approximately 8% increase in the risk of large artery stroke [50].

Another study demonstrated a borderline significant association between O3 exposure and ischemic stroke or TIA [32]. A higher incidence of stroke hospitalization was associated with O3 exposure; this was particularly true for ischemic stroke, and the association was stronger in older age groups [34]. One study found that ambient O3 had different effects in different seasons: It had a positive association with hospital admission for stroke during warm seasons and a negative association during cold seasons [37]. In two-pollutant models, a combination of O3 and a 10 μg/m^3^ increase in NO2 led to a 0.22% increase in the risk of emergency stroke [48]. After other pollutants were included in the model, particularly PM10, the effect of O3 on stroke risk in men remained significant [49].

Interestingly, another study showed that O3 could have a protective effect on stroke patients with coronary atherosclerosis [51].

Black Carbon and Stroke

A number of studies have evaluated the relationship between black carbon (BC) and stroke. One found that BC exposure from local traffic sources was positively associated with stroke incidence. However, no association was found between stroke and BC exposure from residential heating sources [43]. Another study found a significant association between stroke risk and BC exposure due to traffic pollution [40]. However, yet another study found no increased risk of ischemic stroke of any etiology in the 72 hours following exposure to BC. A stratified analysis found an increased risk of ischemic stroke arising from large artery atherosclerosis at two-time points: 24 to 47 hours and 48 to 72 hours after exposure to BC [49].

NO2, NOx, SO2, and Stroke

There may be a significant association between NO2 exposure due to traffic pollution and the risk of stroke [34,40]. One study found a borderline significant relationship between NO2 and the incidence of stroke, specifically ischemic and unspecified stroke; however, the same study found that NO2 exposure was negatively associated with hemorrhagic stroke. That study also found that 10 years of education significantly attenuated the negative impacts of NO2 exposure [52].

One study found a positive association between hospital admission for stroke and exposure to NO2 or SO2; this association was strongest during warm seasons and in individuals over 65 years old. A stratified analysis by season showed that both NO2 and SO2 have stronger associations with stroke admission during warm seasons than cold seasons [53]. Another study found that, during warm seasons, stroke admissions increased on the same day, the previous day, and for a three-day moving average following exposure to NO2; however, during cold seasons, this positive association was only observed on the same day as exposure [53]. NOx from local road traffic is associated with stroke incidence in areas with comparatively low air pollution levels [31]. NO2 and NOX exposure in the previous three days was also found to have a significant association with a higher risk of hemorrhagic stroke in a large cohort of postmenopausal women. This effect was more pronounced in non-obese participants than in obese participants [44].

## Conclusions

Based on the findings of the studies included in this literature review, air pollution is a real global threat. In previous decades, concerns about air pollution were limited to its relationship with cardiovascular and respiratory disorders. Today, however, there is accumulating evidence that there is a strong association between air pollution and CNS disorders. The neurotoxicity of air pollution may impact neuroinflammation, oxidative stress, and cerebral vascular damage through several mechanisms. However, further studies on air pollution and its adverse effects on the CNS are needed. The findings of such studies will support the development of appropriate preventive measures in the future.

## References

[1] (2021). World Health Organization. Air pollution. https://www.who.int/health-topics/air-pollution#tab=tab_1.

[2] Costa LG, Cole TB, Coburn J, Chang YC, Dao K, Roque P (2014). Neurotoxicants are in the air: convergence of human, animal, and in vitro studies on the effects of air pollution on the brain. Biomed Res Int.

[3] Block ML, Elder A, Auten RL (2012). The outdoor air pollution and brain health workshop. Neurotoxicology.

[4] Block ML, Calderón-Garcidueñas L (2009). Air pollution: mechanisms of neuroinflammation and CNS disease. Trends Neurosci.

[5] Kim KH, Kabir E, Kabir S (2015). A review on the human health impact of airborne particulate matter. Environ Int.

[6] Goldenberg MM (2012). Multiple sclerosis review. P T.

[7] GBD 2016 Multiple Sclerosis Collaborators (2019). Global, regional, and national burden of multiple sclerosis 1990-2016: a systematic analysis for the Global Burden of Disease Study 2016. Lancet Neurol.

[8] Heydarpour P, Amini H, Khoshkish S, Seidkhani H, Sahraian MA, Yunesian M (2014). Potential impact of air pollution on multiple sclerosis in Tehran, Iran. Neuroepidemiology.

[9] Lavery AM, Waubant E, Casper TC (2018). Urban air quality and associations with pediatric multiple sclerosis. Ann Clin Transl Neurol.

[10] Gregory AC 2nd, Shendell DG, Okosun IS, Gieseker KE (2008). Multiple sclerosis disease distribution and potential impact of environmental air pollutants in Georgia. Sci Total Environ.

[11] Angelici L, Piola M, Cavalleri T (2016). Effects of particulate matter exposure on multiple sclerosis hospital admission in Lombardy region, Italy. Environ Res.

[12] Bergamaschi R, Cortese A, Pichiecchio A (2018). Air pollution is associated to the multiple sclerosis inflammatory activity as measured by brain MRI. Mult Scler.

[13] Jeanjean M, Bind MA, Roux J, Ongagna JC, de Sèze J, Bard D, Leray E (2018). Ozone, NO(2) and PM(10) are associated with the occurrence of multiple sclerosis relapses. Evidence from seasonal multi-pollutant analyses. Environ Res.

[14] Mehrpour M, Shams-Hosseini NS, Rezaali S, Sahraiian MA, Taki S (2013). Effect of air pollutant markers on multiple sclerosis relapses. Iran J Public Health.

[15] Oikonen M, Laaksonen M, Laippala P (2003). Ambient air quality and occurrence of multiple sclerosis relapse. Neuroepidemiology.

[16] Roux J, Bard D, Le Pabic E (2017). Air pollution by particulate matter PM(10) may trigger multiple sclerosis relapses. Environ Res.

[17] Vojinović S, Savić D, Lukić S, Savić L, Vojinović J (2015). Disease relapses in multiple sclerosis can be influenced by air pollution and climate seasonal conditions. Vojnosanit Pregl.

[18] Ashtari F, Esmaeil N, Mansourian M, Poursafa P, Mirmosayyeb O, Barzegar M, Pourgheisari H (2018). An 8-year study of people with multiple sclerosis in Isfahan, Iran: association between environmental air pollutants and severity of disease. J Neuroimmunol.

[19] Tateo F, Grassivaro F, Ermani M, Puthenparampil M, Gallo P (2019). PM2.5 levels strongly associate with multiple sclerosis prevalence in the Province of Padua, Veneto Region, North-East Italy. Mult Scler.

[20] Bergamaschi R, Monti MC, Trivelli L, Mallucci G, Gerosa L, Pisoni E, Montomoli C (2021). PM(2.5) exposure as a risk factor for multiple sclerosis. An ecological study with a Bayesian mapping approach. Environ Sci Pollut Res Int.

[21] Türk Börü Ü, Bölük C, Taşdemir M, Gezer T, Serim VA (2020). Air pollution, a possible risk factor for multiple sclerosis. Acta Neurol Scand.

[22] Yuchi W, Sbihi H, Davies H, Tamburic L, Brauer M (2020). Road proximity, air pollution, noise, green space and neurologic disease incidence: a population-based cohort study. Environ Health.

[23] Bai L, Burnett RT, Kwong JC (2018). Long-term exposure to air pollution and the incidence of multiple sclerosis: a population-based cohort study. Environ Res.

[24] Palacios N, Munger KL, Fitzgerald KC, Hart JE, Chitnis T, Ascherio A, Laden F (2017). Exposure to particulate matter air pollution and risk of multiple sclerosis in two large cohorts of US nurses. Environ Int.

[25] Chen H, Kwong JC, Copes R (2017). Living near major roads and the incidence of dementia, Parkinson's disease, and multiple sclerosis: a population-based cohort study. Lancet.

[26] Zhao CN, Xu Z, Wu GC (2019). Emerging role of air pollution in autoimmune diseases. Autoimmun Rev.

[27] Cortese A, Lova L, Comoli P (2020). Air pollution as a contributor to the inflammatory activity of multiple sclerosis. J Neuroinflammation.

[28] Kumar P, Saini S, Khan S, Surendra Lele S, Prabhakar BS (2019). Restoring self-tolerance in autoimmune diseases by enhancing regulatory T-cells. Cell Immunol.

[29] Niu Z, Liu F, Yu H, Wu S, Xiang H (2021). Association between exposure to ambient air pollution and hospital admission, incidence, and mortality of stroke: an updated systematic review and meta-analysis of more than 23 million participants. Environ Health Prev Med.

[30] Guo P, Wang Y, Feng W (2017). Ambient air pollution and risk for ischemic stroke: a short-term exposure assessment in South China. Int J Environ Res Public Health.

[31] Korek MJ, Bellander TD, Lind T (2015). Traffic-related air pollution exposure and incidence of stroke in four cohorts from Stockholm. J Expo Sci Environ Epidemiol.

[32] Lisabeth LD, Escobar JD, Dvonch JT (2008). Ambient air pollution and risk for ischemic stroke and transient ischemic attack. Ann Neurol.

[33] Zhang R, Jiang Y, Zhang G, Yu M, Wang Y, Liu G (2021). Association between short-term exposure to ambient air pollution and hospital admissions for transient ischemic attacks in Beijing, China. Environ Sci Pollut Res Int.

[34] Shin S, Burnett RT, Kwong JC (2019). Ambient air pollution and the risk of atrial fibrillation and stroke: a population-based cohort study. Environ Health Perspect.

[35] Amini H, Dehlendorff C, Lim YH (2020). Long-term exposure to air pollution and stroke incidence: a Danish Nurse cohort study. Environ Int.

[36] Zeng W, Zhang Y, Wang L (2018). Ambient fine particulate pollution and daily morbidity of stroke in Chengdu, China. PLoS One.

[37] Huang K, Liang F, Yang X (2019). Long term exposure to ambient fine particulate matter and incidence of stroke: prospective cohort study from the China-PAR project. BMJ.

[38] Wellenius GA, Schwartz J, Mittleman MA (2005). Air pollution and hospital admissions for ischemic and hemorrhagic stroke among medicare beneficiaries. Stroke.

[39] Zhang R, Liu G, Jiang Y (2018). Acute effects of particulate air pollution on ischemic stroke and hemorrhagic stroke mortality. Front Neurol.

[40] Wellenius GA, Burger MR, Coull BA (2012). Ambient air pollution and the risk of acute ischemic stroke. Arch Intern Med.

[41] Qiu H, Sun S, Tsang H, Wong CM, Lee RS, Schooling CM, Tian L (2017). Fine particulate matter exposure and incidence of stroke: a cohort study in Hong Kong. Neurology.

[42] Lin H, Tao J, Du Y (2016). Differentiating the effects of characteristics of PM pollution on mortality from ischemic and hemorrhagic strokes. Int J Hyg Environ Health.

[43] Ljungman PLS, Andersson N, Stockfelt L (2019). Long-term exposure to particulate air pollution, black carbon, and their source components in relation to ischemic heart disease and stroke. Environ Health Perspect.

[44] Sun S, Stewart JD, Eliot MN (2019). Short-term exposure to air pollution and incidence of stroke in the Women's Health Initiative. Environ Int.

[45] Vivanco-Hidalgo RM, Avellaneda-Gómez C, Dadvand P (2019). Association of residential air pollution, noise, and greenspace with initial ischemic stroke severity. Environ Res.

[46] Vivanco-Hidalgo RM, Wellenius GA, Basagaña X (2018). Short-term exposure to traffic-related air pollution and ischemic stroke onset in Barcelona, Spain. Environ Res.

[47] Han MH, Yi HJ, Kim YS, Kim YS (2015). Effect of seasonal and monthly variation in weather and air pollution factors on stroke incidence in Seoul, Korea. Stroke.

[48] Tang C, Chen Y, Song Q (2021). Short-term exposure to air pollution and occurrence of emergency stroke in Chongqing, China. Int Arch Occup Environ Health.

[49] Henrotin JB, Besancenot JP, Bejot Y, Giroud M (2007). Short-term effects of ozone air pollution on ischaemic stroke occurrence: a case-crossover analysis from a 10-year population-based study in Dijon, France. Occup Environ Med.

[50] Suissa L, Fortier M, Lachaud S, Staccini P, Mahagne MH (2013). Ozone air pollution and ischaemic stroke occurrence: a case-crossover study in Nice, France. BMJ Open.

[51] Chen C, Liu X, Wang X, Qu W, Li W, Dong L (2020). Effect of air pollution on hospitalization for acute exacerbation of chronic obstructive pulmonary disease, stroke, and myocardial infarction. Environ Sci Pollut Res Int.

[52] Andersen ZJ, Kristiansen LC, Andersen KK (2012). Stroke and long-term exposure to outdoor air pollution from nitrogen dioxide: a cohort study. Stroke.

[53] Huang F, Luo Y, Tan P (2017). Gaseous air pollution and the risk for stroke admissions: a case-crossover study in Beijing, China. Int J Environ Res Public Health.

